# To See or Not to See: Investigating Detectability of Ganges River Dolphins Using a Combined Visual-Acoustic Survey

**DOI:** 10.1371/journal.pone.0096811

**Published:** 2014-05-07

**Authors:** Nadia I. Richman, James M. Gibbons, Samuel T. Turvey, Tomonari Akamatsu, Benazir Ahmed, Emile Mahabub, Brian D. Smith, Julia P. G. Jones

**Affiliations:** 1 Institute of Zoology, Zoological Society of London, Regent’s Park, London, United Kingdom; 2 School of Environment, Natural Resources and Geography, Bangor University, Gwynedd, United Kingdom; 3 National Research Institute of Fisheries Engineering, Fisheries Research Agency, Kamisu, Ibaraki, Japan; 4 Department of Zoology, University of Chittagong, Chittagong, Bangladesh; 5 Bangladesh Cetacean Diversity Project, Wildlife Conservation Society, Khulna, Bangladesh; 6 Ocean Giants Program, Wildlife Conservation Society, Bronx, New York, United States of America; Università degli Studi di Napoli Federico II, Italy

## Abstract

Detection of animals during visual surveys is rarely perfect or constant, and failure to account for imperfect detectability affects the accuracy of abundance estimates. Freshwater cetaceans are among the most threatened group of mammals, and visual surveys are a commonly employed method for estimating population size despite concerns over imperfect and unquantified detectability. We used a combined visual-acoustic survey to estimate detectability of Ganges River dolphins (*Platanista gangetica gangetica*) in four waterways of southern Bangladesh. The combined visual-acoustic survey resulted in consistently higher detectability than a single observer-team visual survey, thereby improving power to detect trends. Visual detectability was particularly low for dolphins close to meanders where these habitat features temporarily block the view of the preceding river surface. This systematic bias in detectability during visual-only surveys may lead researchers to underestimate the importance of heavily meandering river reaches. Although the benefits of acoustic surveys are increasingly recognised for marine cetaceans, they have not been widely used for monitoring abundance of freshwater cetaceans due to perceived costs and technical skill requirements. We show that acoustic surveys are in fact a relatively cost-effective approach for surveying freshwater cetaceans, once it is acknowledged that methods that do not account for imperfect detectability are of limited value for monitoring.

## Introduction

Estimates of abundance, trends over time, and distribution are all important for conservation management of threatened species [1]–[3]. To reliably estimate population size or habitat use, detectability, and how it may vary with time and space, must be estimated and accounted for [4]. Freshwater cetaceans are one of the most threatened groups of mammals on earth. Accurate assessment of population size, trends and distribution are therefore of great importance [5]. However, limited resources and a lack of robust survey methods mean that basic information on river dolphin status and trends is lacking across large parts of their ranges.

The use of methods typically used for monitoring marine cetaceans is largely precluded for freshwater cetaceans due to constraints arising from survey conditions in river systems, and from differences in freshwater cetacean morphology and surfacing behaviour [6]. Distance sampling using a visual line transect is commonly used to survey marine cetacean species including Sperm whales (*Physeter macrocephalus*) [7], Killer whales (*Orcinus orca*) [8], and Vaquita (*Phocoena sinus*) [9]. This method has been attempted with freshwater cetaceans, e.g. Ganges River dolphins (*Platanista gangetica gangetica*) [10], Yangtze Finless porpoises (*Neophocaena asiaeorientalis asiaeorientalis*) [11], and Amazon River dolphins (*Inia geoffrensis*) [12]) (Table 1). However, bathymetrical constraints in river systems mean that survey vessels usually cannot follow transect lines that are distributed randomly with respect to the distribution of cetaceans, violating a key assumption of distance sampling [13]. Mark-recapture using photo-identification has also been used to estimate the abundance of some freshwater cetaceans, such as Irrawaddy dolphins (*Orcaella brevirostris*) [14], [15]. However, the exceptionally small dorsal fin (or lack of one altogether in finless porpoises) and rapid surfacing behaviour of other freshwater cetacean species limits the feasibility of photo-identification, making mark-recapture generally impractical [6].

**Table 1:** A summary of methods used for estimating abundance of freshwater cetaceans over the last twenty years.

Method	Species	Advantages	Disadvantages	Examples
**Distance sampling with visual line transect**	Amazon River dolphin, Ganges River dolphin, Yangtze Finless porpoise	1. Can account for imperfect detectability.	1. Difficult or impossible to meet the assumption that dolphin distribution is random relative to the transect line because: a) cannot place a random transect line as vessels are constrained to following a deep navigable channel or shipping lane; b) dolphin distribution is not random and may be confined to the same deep navigable channel as vessels, or clustered at river banks.	[11], [12], [19]
**Mark-recapture with photo-identification**	Irrawaddy dolphin, Amazon River dolphin, Ganges River dolphin, Yangtze River dolphin	1. Can account for imperfect detectability.	1. Difficult to match individuals for species with limited recognisable markings and short surfacing times.	[14], [15], [75], [76], [77], [78]
			2. Possible invalidation of the assumption of population closure between sampling periods, due to length of time required to obtain enough photographs in one sampling period.	
			3. Requires a good photographer and expensive equipment.	
**Single observer-team visual survey**	Ganges River dolphin, Yangtze Finless porpoise, Amazon River dolphin	1. Requires little training or expertise.	1. Cannot account for imperfect detectability.	1. [16], [17], [18], [19], [20], [21], [22], [79], [80]
**Double observer-team visual survey**	Ganges River dolphin, Yangtze Finless porpoise, Irrawaddy dolphin, Amazon River dolphin	1. Can account for imperfect detectability.	1. Requires a vessel large enough to accommodate two independent teams.	[23]
			2. Impossible in shallow rivers.	
			3. Extra cost associated with a larger survey vessel and extra team.	
**Tandem-vessel visual survey**	Indus River dolphin	1. Can account for imperfect detectability.	1. Cost of an additional survey vessel.	[24]
**Combined visual-acoustic survey**	Yangtze Finless porpoise	1. Can account for imperfect detectability.	1. Requires expensive equipment.	[25]
		2. A double-observer platform is not needed and so the survey can be carried out in small boats.	2. Specialist expertise needed to analyse the data.	
		3. The small boats needed can survey shallow rivers as well as larger rivers.	3. Acoustic detection range may be limited in environments with high levels of unwanted noise e.g. high density vessel traffic.	
		4. Acoustic surveys yield higher detection probabilities than visual methods, so can provide more precise estimates of abundance.		

Surveys of freshwater cetaceans often rely on counts from a single observer-team [16]–[22] on a boat following the thalweg or deepest area of the river channel (Table 1). Estimates of abundance from single observer-team visual surveys reflect a minimum population size because an unknown number of animals remains undetected [6]. Detectability of cetaceans is affected by two sources of bias: availability and perception [6]. Because of high turbidity, cetaceans in rivers are typically only available for detection when at the water surface. Availability for detection is therefore determined by dive times [6] and group size, with larger groups being more detectable than smaller groups [23]. Even if a cetacean is available for detection at the water surface, it may still go undetected due to perception bias resulting from inattention, observer fatigue, visual barriers (e.g. ships, bridge pilings and channel meanders), distance from observers, and poor sighting conditions [6]. Independent observer teams, either on the same vessel (i.e. double observer-team visual surveys) [23] or on separate vessels following one another (i.e. tandem-vessel visual surveys) [11], [24], can be used to estimate detectability related to perception bias with closed capture-recapture models. However, many rivers are too shallow to accommodate a survey vessel large enough to accommodate independent teams, and tandem-vessel visual surveys can be problematic as it can be difficult to distinguish individual groups and therefore match detections made by the front and rear vessels, especially at higher densities [24]. These methods also do not account for availability bias.

An alternative (or supplementary) approach to visual surveys is the use of passive acoustic survey methods which allow cetaceans to be detected underwater, thus increasing their detectability assuming the animals are vocalizing and within detection range [7], [25]. Small cetaceans, especially species occurring in turbid freshwater environments, are particularly good candidates for acoustic detection because they must vocalise frequently for navigation due to the poor visibility and complexity of their environment [26], [27]. Acoustic methods have been employed in a number of studies of Yangtze Finless porpoises and Ganges River dolphins looking at underwater behaviour [28], [29], echolocation characteristics [30]–[32] and abundance estimation [25], [33], [34]. However, despite their demonstrated efficacy at improving detectability of animals, uptake of acoustic surveys has been slow due to perceived costs and technical skill requirements [35].

The Ganges River dolphin is listed as Endangered in the IUCN Red List of Threatened Species [36]. It is regarded as a high conservation priority due to the range and magnitude of threats it faces, and its unique evolutionary history as a relict lineage [37]. Ganges River dolphins are in widespread decline across the South Asian subcontinent due to bycatch by fishers, intentional killing for meat and oil, habitat loss, and probably pollution and boat collisions [38]–[42]. Identification of robust, cost-effective methods to assess population sizes and trends is therefore an important priority. We used a combined visual-acoustic survey to investigate the factors affecting visual detectability of Ganges River dolphins, and make recommendations for the design of future surveys of freshwater cetaceans. We explore how detectability affects power to detect population trends, and the relative costs of different survey methods.

## Materials and Methods

### Study Site

In January and February 2012, surveys were carried out in three interconnected rivers and one canal in southern Bangladesh (Chittagong district) (see Figure S1 in supplementary information). Surveys covered a 20 km section of the Halda River, a 45 km section of the Sangu River, and the entire Karnaphuli River (75 km) and Shikalbaha-Chandkhali Canal (29 km). The Karnaphuli River was divided into the Upper Karnaphuli (the 47 km river section upstream of Kalurghat Bridge) and Lower Karnaphuli (the 28 km river section downstream of Kalurghat Bridge including Chittagong Port) because of differences between the two sections: the Upper Karnaphuli runs through plantations (teak and tea), agricultural land and small villages and has very low densities of vessel traffic, while the Lower Karnaphuli is considerably wider and the riverbanks are dominated by a ship-breaking yard, a naval port, and Chittagong city. Waterways varied in width from 35 to 2,300 m, with a mean of 607 m (SD = 449). Mean water depth in the approximate thalweg ranged from 5.4 m (SD = 5.2) in the Sangu, to 8.4 m (SD = 4.4) in the Lower Karnaphuli. Due to shallow water depth, the survey vessel was regularly constrained to following the river thalweg. The research was carried out under a research permit issued to the lead author from the Ministry of Environment and Forest, Government of the People’s Republic of Bangladesh.

### Pilot Surveys

In January 2012, two pilot surveys were carried out to identify dolphin distribution, and determine survey strip width based on the visual range of observers. Both surveys were carried out under favourable sighting conditions [6]. Waterways shallower than 50 cm in depth were excluded from the survey, as the pilot surveys and prior four months of field experience found no dolphins at depths this shallow. The pilot phase also included a study of dolphin dive time (see [6] for an outline of the method) based on six single animals and two groups of three animals.

### Visual and Acoustic Survey

The combined visual-acoustic survey was carried out in February 2012, the low-water season, when sighting conditions are most favourable [6]. Surveys were carried out using a local motorised boat with a single observer-team during daylight hours. The observer-team consisted of a left, right, and central observer and a data recorder. All observers were trained to maximize consistency in distance estimation: observers were asked to estimate distance by eye using objects such as boats and bridge pilings, which were then compared to the distance measured by the lead observer using a global positioning system (GPS). Observers were positioned on the roof of the vessel at an eye height of 2.5–3.0 m above water level, and were rotated with two resting observers every 30 minutes to avoid fatigue [6]. Left and right observers searched from 90^o^ off the left and right beam to 10^o^ beyond the bow using Olympus 10×50 binoculars and the naked eye. The central observer used the naked eye to search a 20^o^ cone in front of the bow (10^o^ either side of the transect line).

Weather conditions (sun glare, wind, and rain/fog) and survey effort were recorded at 30 minute intervals, or whenever conditions changed, on a scale of 0–2 as described by [23]. Scores were then summed to give a cumulative score on a scale of 0–6 (0 = excellent conditions, 6 = poor conditions). When a dolphin was sighted, the data recorder noted the latitude/longitude (using Garmin eTrex Summit HC Global Positioning System), estimates of distance and relative angle from the transect line to the sighting, time, vessel speed, group size as best/high/low estimates, and observer name. A group was defined as all individuals within 100 m of each other. All group size estimates were made in passing mode (i.e. survey vessel continues along the transect line) [43].

**Figure 1:**
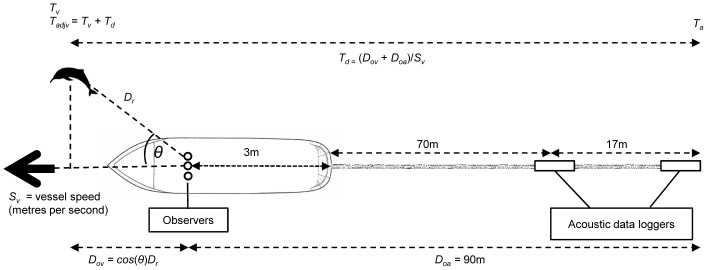
Schematic of the visual and acoustic survey set-up, with details of measurements taken for matching detections. Illustration of the visual and acoustic survey set-up, and measurements necessary for matching visual and acoustic detections including: time of visual detection (*T_v_*), time of acoustic detection (*T_a_*), time difference between time of visual detection and time of acoustic detection (*T_d_*), radial distance of dolphin from observer (*D_r_*), adjusted visual time (*T_adjv_*), straight distance between dolphin and observer (*D_ov_*), vessel speed (*S_v_*), and distance between furthest acoustic data logger and observer (*D_oa_*).

A simultaneous acoustic survey from the same survey vessel was carried out using a towed hydrophone array system consisting of two stereo pulse event data loggers (A-tags: ML200-AS2, Marine Micro Technology, Saitama, Japan). Two data loggers were towed astern of the vessel on an 87 m long rope, with one positioned at 70 m and the other at 87 m. Each data logger consisted of two hydrophones separated by 19 cm (Figure 1). Hydrophone sensitivity of the data logger was set to −200dB/V at 130 kHz (100–160 kHz within −5dB band) which is close to the vocalisation frequency of the Ganges River dolphin [44], [45].

To minimise the effect of availability bias, boat speed must be slow enough to allow dolphins to surface at least once within the visual range of observers, but fast enough to minimise the chance of a dolphin swimming past the boat twice (i.e. “double counting”). To estimate the visual range of observers we plotted a frequency distribution of the radial sighting distances of detections during pilot surveys of the Sangu, Halda and Upper Karnaphuli rivers. Sighting frequencies fell off rapidly beyond 200 m, and so this distance was used to define the visual range of observers. Mean dive time for the six single animals was 68 seconds (*n* = 192 surfacings, 95% CI = 64–71) and 41 seconds for the two groups of three (*n* = 245 surfacings, 95% CI = 38–44). We selected 10 km/hr as the boat speed for the survey; at this speed it would take 72 seconds for the boat to travel 200 m, allowing single animals to surface at least once within the visual range of observers. While mean estimates of dive time vary across studies [23], [24], [46], [47], observers typically have an unobstructed view of the river surface further than 200 m ahead of the vessel and so still have the opportunity to detect surfacings of longer diving animals. Dive time in Ganges River dolphins can be affected by activity type (e.g. feeding, resting, travelling) [47] which is affected by time of day and tidal state [48]. Surveys of each river were carried out at the same tidal state (flood tide and high tide slack) and time of day (8 am–noon), thereby controlling for dolphin activity as much as possible. In another recent survey of Ganges River dolphins [23], the authors assumed that at a mean boat speed of 10 km/hr availability bias was unlikely to significantly negatively affect visual detectability. To reduce perception bias, observers were rotated with off-duty observers, thereby minimising observer fatigue; we surveyed a fixed strip width of 400 m (or less depending on channel width) based on a 200 m observer visual range either side of the transect line; and all surveys were carried out in very good to excellent sighting conditions with a cumulative score never exceeding 1.

Acoustic detection range depends on the sound pressure level emitted by vocalising animals [25]. Dolphin detectability by acoustic data loggers is reduced with increasing distance, as sound pressure level from vocalising dolphins becomes lower than the detection threshold of the data loggers [25]. Acoustic detection of dolphins can also be negatively affected by high levels of background noise (e.g. from motorised vessels). An acoustic survey of Yangtze Finless porpoises using the same data loggers as used in this study calculated an effective acoustic detection distance of 300 m from the transect line [25], beyond which acoustic detectability was found to decline significantly. As source levels from Ganges River dolphins and Yangtze Finless porpoises are comparable [27], we assumed that the 200 m detection range either side of the transect line used for the visual survey would be sufficient for acoustic detection.

### Matching Acoustic and Visual Detections

Ganges River dolphin vocalisations were visualized using an automated off-line software developed in Igor Pro 6.22A [49]. Dolphin vocalisations form predictable patterns in inter-click interval and sound pressure level that can be differentiated from random background noise [50]. In environments where there is considerable background noise, estimation of acoustically detected individuals is problematic as it is difficult to distinguish dolphin click trains from noise. In addition, it is increasingly difficult to distinguish individual click trains from one another when animals are very close to one another. To determine the likelihood of overestimating or underestimating the number of acoustically detected individuals we examined the level of background noise to assess the potential for incorrect identification or missing of click trains. We also used the method described in Akamatsu et al. [25] in which we compare acoustic and visual group sizes for matched detections, to look for evidence of underestimation of acoustically detected individuals.

Incorrect matching of visual and acoustic detections is potentially the greatest source of error in abundance estimation during combined visual-acoustic surveys [51]. Studies of marine cetaceans employing combined visual-acoustic surveys typically match visual and acoustic detections using the location of each at time of detection, and allowing for movement of individual animals based on knowledge of species movement patterns in response to survey vessels e.g. [8]. However, little is known about the response of freshwater cetaceans to the presence of survey vessels. Akamatsu et al. [33] proposed a multimodal detection model for matching visual and acoustic detections of Ganges River dolphins based on species dive time and time interval between vocalisations. While several published dive time estimates are available for this species [23], [24], [46], [47], along with the data we collected during this study (see above), there is both considerable variation in estimates across studies and also wider uncertainty regarding the factors (e.g. ecological, behavioural) affecting dive time. Based on these concerns, we use a distance window for matching detections, similar to the time window approach described in [25] which requires no assumptions on species dive time. We opted to use a distance window for matching detections rather than a time window, as time windows rely on the assumption that boat speed remains constant throughout the survey.

**Figure 2:**
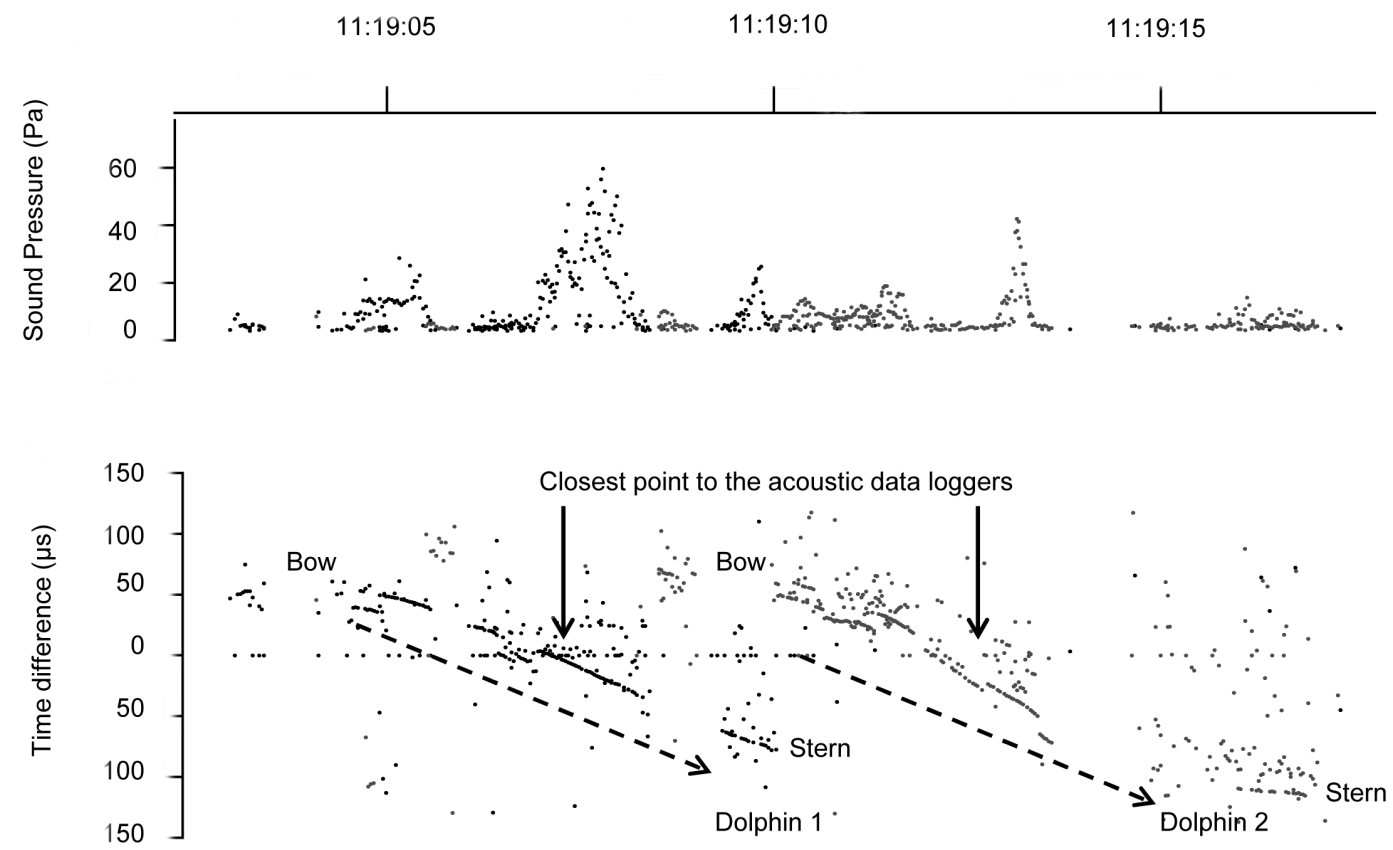
Patterns in sound pressure level and inter-click interval of Ganges River dolphin clicks. Trace of click trains from two Ganges River dolphins as they pass in a bow-to-stern direction illustrated using the time difference (µs) in inter-click interval (bottom image) and sound pressure level (top image).

A key assumption of matching visual and acoustic detections is that animals are first detected by visual observers ahead of the vessel, and then by acoustic data loggers astern of the vessel. To ensure that dolphins could not swim in a stern-to-bow direction, boat speed should be faster than the swim speed of Ganges River dolphins. While no studies have investigated the maximum swim speed of this species, a recent study recorded individuals travelling at an average of 3.5 km/hr [28], similar to that found for other freshwater cetaceans (Amazon River dolphin, typically <5.5 km/hr; Yangtze River dolphin (*Lipotes vexillifer*), 1.5–3 km/hr) [52]–[54]. We validated this assumption by visualising the shape of the click train that indicates the direction in which dolphins passed the acoustic data logger (Figure 2). All click trains ran from a positive to negative angle in inter-click interval, indicating that animals passed the data loggers in a bow-to-stern direction. The time delay between when the sound source reaches the two hydrophones can be used to calculate the conical bearing angle to the sound source with a resolution of 271 ns [55]. The time at which a dolphin was detected was defined as the point when the signal arrival time between the two hydrophones was zero or closest to zero, indicating that the dolphin was closest to the data logger [25]. This method allows us to count the number of vocalising animals rather than the number of vocalisations.

Another fundamental assumption of closed population capture-recapture studies is that animals are not lost from the study area (i.e. 400 m survey strip) between visual and acoustic detection. If dolphins avoid or are attracted to survey vessels this may result in the loss or gain of animals between detection events. However, an independent study found no evidence of vessel avoidance or attraction in the closely related Indus River dolphin (*Platanista gangetica minor*) [24]. In addition to which, only 9% of the length of all water ways exceeded the strip width and so there was little opportunity for animals to leave the study area.

We first accounted for the time difference 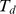 between both visual and acoustic detections, given that visual detections are made ahead of the vessel and acoustic detections are made astern of the vessel (Figure 1). To calculate the time difference we calculated the distance between observers and the point of visual detection along the transect line 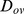, where 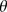 represents the relative angle of the visual detection from the transect line and 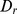 is the radial distance of the animal from the observers. To obtain 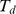 we added 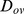 to the distance between observers and acoustic data loggers 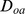 and divided by the GPS recorded vessel speed at the time of visual detection 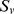:
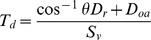


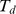
 was then added to the original time of visual detection 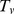 to give the adjusted time of visual detection 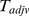, which accounts for the time lag between visual and acoustic detection:



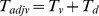



If the dolphin did not move between visual and acoustic detection then the difference between 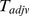 and acoustic detection time 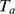 is zero. However, if the dolphin swam towards the vessel then 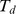 is decreased and if it swam away from the vessel then 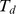 is increased, resulting in a negative or positive value between 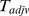 and 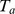. To match acoustic and visual detections, we applied a distance window to each 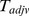. The window ran in both a negative and positive direction to account for dolphins that swam either towards or away from observers between 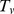 and 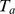. Only a single 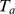 could be matched to a single 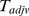; where more than one *T_a_* fell within a distance window, the one closest to 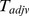 was considered a match and the other was considered unmatched. However, where animals were detected in a group of two or more, only a single distance measurement was taken to the centre of the group. As groups were defined by all animals within 100 m of each other, any individual detected within a group was matched using the defined distance window plus an additional 100 m. To determine a distance window, we calculated the distance difference between 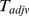 and the closest 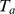. We plotted a cumulative frequency distribution of matched 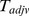 and 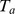 at fifty metre intervals and selected a threshold distance by visual inspection of the frequency distribution.

### Calculating Detectability

Detection probabilities were estimated using mark-recapture analysis, where visual observation is considered a mark and acoustic detection is considered a recapture [48]. A Lincoln-Peterson estimator was used and detectability was calculated for each river. This approach is appropriate because the population was closed between samples and we assume that all individuals had an equal chance of being detected.

By re-arrangement of the standard Lincoln-Petersen estimator, we calculated visual and acoustic survey detection probabilities (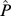) and 95% confidence intervals (95% CI) using the following equations:
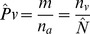





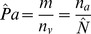
and 95% confidence intervals (95% CI):



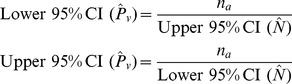





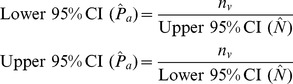
where abundance (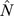) is:



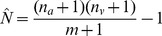
where 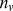 is the number of animals detected visually, 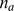 is the number of animals detected acoustically, and 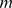 is the number of matched detections.

### Power to Detect Population Trends

For a population to be considered Critically Endangered under IUCN criterion A, a minimum decline of 80% over three generation lengths has to occur. Assuming three generations is 60 years for the Ganges River dolphin (see [36] for details) and a constant rate of decline, this is equivalent to a 2.75% annual decline. To illustrate differences in power between a single observer-team visual survey and a combined visual-acoustic survey, we estimated the number of repeat surveys required to detect change in a population declining at this rate over a 10 year interval (i.e. a 24% decline). Abundance (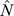) and variance (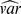) for the 400 m survey strip detailed in this study were estimated in MARK [56] using the Chapman-modified Lincoln Petersen estimator. The 400 m survey strip population estimate does not represent an overall estimate for the entire study area. Wide river width in the Lower Karnaphuli meant the channel had to be split into two strips that were surveyed simultaneously, one with a combined visual-acoustic survey and one with a single observer-team visual survey. An overall population estimate will require the development of correction factors to account for animals missed in sections where there was no acoustic effort.

The CV for the single observer-team visual survey was calculated using the mean and standard deviation of the two pilot visual surveys and the main visual survey, and for the combined visual-acoustic survey using the CV of the 400 m survey strip abundance estimate. The probability of committing a Type 1 error (α) was set to 0.05, and power (β) to 80%. All analyses were carried out in TRENDS version 3 [57].

### Investigating Factors Affecting Visual Detection of River Dolphins

We used a generalized linear model with a binomial error term to model the effect of potential predictors on visual detectability of dolphins. The response was modelled as a binary factor where acoustic detections were either matched with a visual detection {1} or unmatched {0} (n = 110). Predictor variables were observer experience (binary factor coded as: {0} inexperienced, i.e. having no prior cetacean survey experience, or {1} experienced, i.e. having carried out five or more prior surveys), and available observation distance (continuous factor), and the interaction. Ganges River dolphins are known to occur in higher concentrations at meanders [58], but these features can temporarily block the view of the following river section. We modelled available observation distance as the distance between the meander and the dolphin when perpendicular to the survey vessel. Based on our mean estimates of dive time and a boat speed of 10 km/hr, dolphins located less than 200 m from a meander may never surface before the vessel passes by, therefore never becoming available for visual detection. Because ships can create sighting obstructions, we excluded data from the Lower Karnaphuli due to the high density of cargo ships in this region. Variables such as river width, sighting conditions, and observer effort were not included the model as they were controlled for in the survey design. Spearman’s rank correlation was used to test for collinearity between variables.

We developed a global model containing available observation distance and observer experience. A candidate set of eight models was developed *a priori* and fitted in R 3.0.1 [59]. Models were ranked according to Akaike’s information criterion (AIC), and model selection was based on Δ*_i_* (the difference in AIC between model *i* and the minimum AIC for the model set). Where there were models with Δ*_i_* <2, model averaging was used to estimate coefficients as there was no clear support for a single model [60]. We used the model-averaged results to predict visual detectability at available observation distances ranging from 0 to 2,100 metres.

### Cost Analysis

We compared set-up and daily costs for four survey methods (a single observer-team visual survey, a double observer-team visual survey, a tandem-vessel visual survey, and a combined visual-acoustic survey), and calculated the length of time required for each method to exceed a combined visual-acoustic survey in overall cost (i.e. sum of capital and daily running costs). Neither the tandem-vessel visual survey nor double observer-team visual survey were carried out during our field research, but costs for each of these two methods could be calculated from our own single observer-team visual survey. A number of costs were common to each method, but may have differed in quantity. The only cost exclusive to a particular method was the towed hydrophone array system necessary for the acoustic survey. All staff, boat, food and water, and printing costs were based on local Bangladeshi rates but presented in 2013 US dollars using an exchange rate of 1 USD = 79.8 Bangladeshi Taka [61].

## Results

### Visual and Acoustic Detections

We obtained a total of 114 visual detections and 159 acoustic detections. Ninety five percent of visual detections were within 100 m perpendicular distance of the transect line, and 100% were within 200 m. Unfortunately due to failure of an acoustic data logger, acoustic distance information was only available for the first two days of the survey (Halda, Lower Karnaphuli and Sangu rivers). However, of the acoustic detections with distance information, 99% were within 200 m perpendicular distance of the transect line (see Figure S2 in supplementary information).

### Matching Detections

Based on levels of background noise and the comparison of acoustic and visual group sizes, we conclude that our count of acoustic individuals from click train patterns is accurate. There was very little background noise (especially from major broadband sources such as snapping shrimp), and so it is unlikely that the number of click trains was overestimated or underestimated. The comparison of group sizes for matched detections also suggests that the number of acoustically detected individuals was not underestimated. In 74% of cases, numbers of visual and acoustic detections for each matched distance window were equal in size. Of the 26% of matches where the number of detections differed, in most cases (78%) the number of acoustic detections was higher than the number of visual.

**Figure 3:**
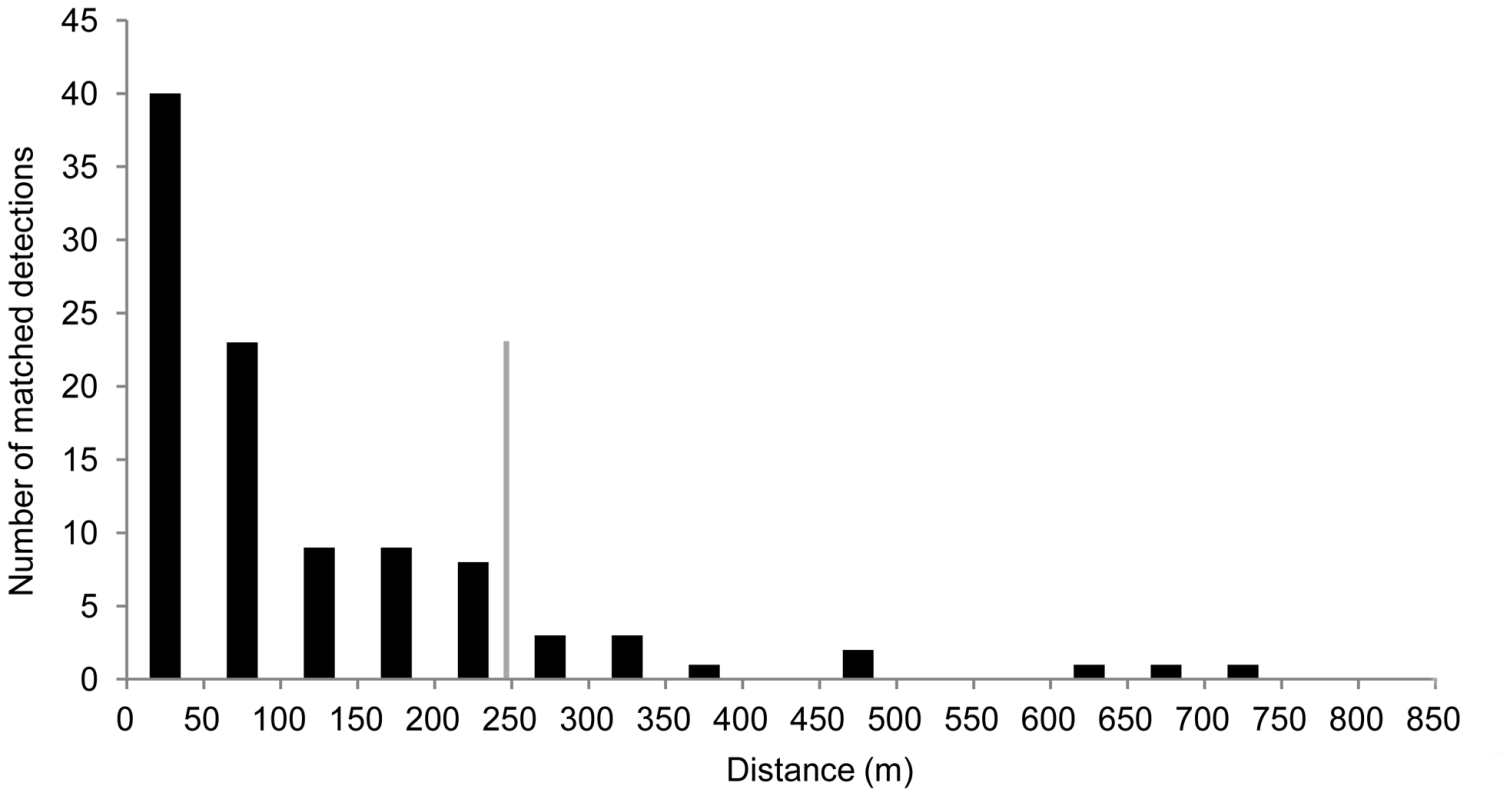
Distribution of potential matched visual and acoustic detections at distance windows from 0–899 m. Frequency of numbers of matched visual and acoustic detections at 50-off point (249 metres) used to match visual and acoustic detections.

Matches were largely unambiguous as the majority of visual detections (56%) and acoustic detections (64%) were of single animals, separated by mean distances ranging from 1.3 km (95% CI = 0.9–1.7 km) in the Halda River, to 11.8 km (95% CI = 7.6–16.2 km) in the Upper Karnaphuli River. There were 102 possible matches, of which 65% occurred within 100 m of each other and 90% occurred within 200 m of each other (Figure 3), supporting our assumption that animals moved relatively small distances between visual and acoustic detection. Based on visual inspection of the frequency distribution of number of visual and acoustic matches over distance, we selected a minimum distance threshold of 249 m for matching single dolphins, but allowed for movement of up to 349 m for group sizes of two or more animals because distance estimates could be out by up to 100 m. Of the 102 possible matches, 91 fell within these distance thresholds, leaving a total of 72 unmatched acoustic detections and 23 unmatched visual detections. Of the unmatched visual detections, 20 were located in the Lower Karnaphuli and Sangu rivers.

Visual detection of dolphins at considerable distances ahead of the survey vessel can increase the distance threshold required for matching, as a longer time frame between visual and acoustic detection means that dolphins have more time to move. However, 85% of visual detections were made within 300 m travelling distance along the transect line. Based on a mean boat speed of 9.1 km/hr and an estimated dolphin swimming speed of 5.5 km/hr, dolphins could have moved up to 358 m between visual and acoustic detection; our detection thresholds more than account for this potential movement.

### Detection Probabilities

**Figure 4:**
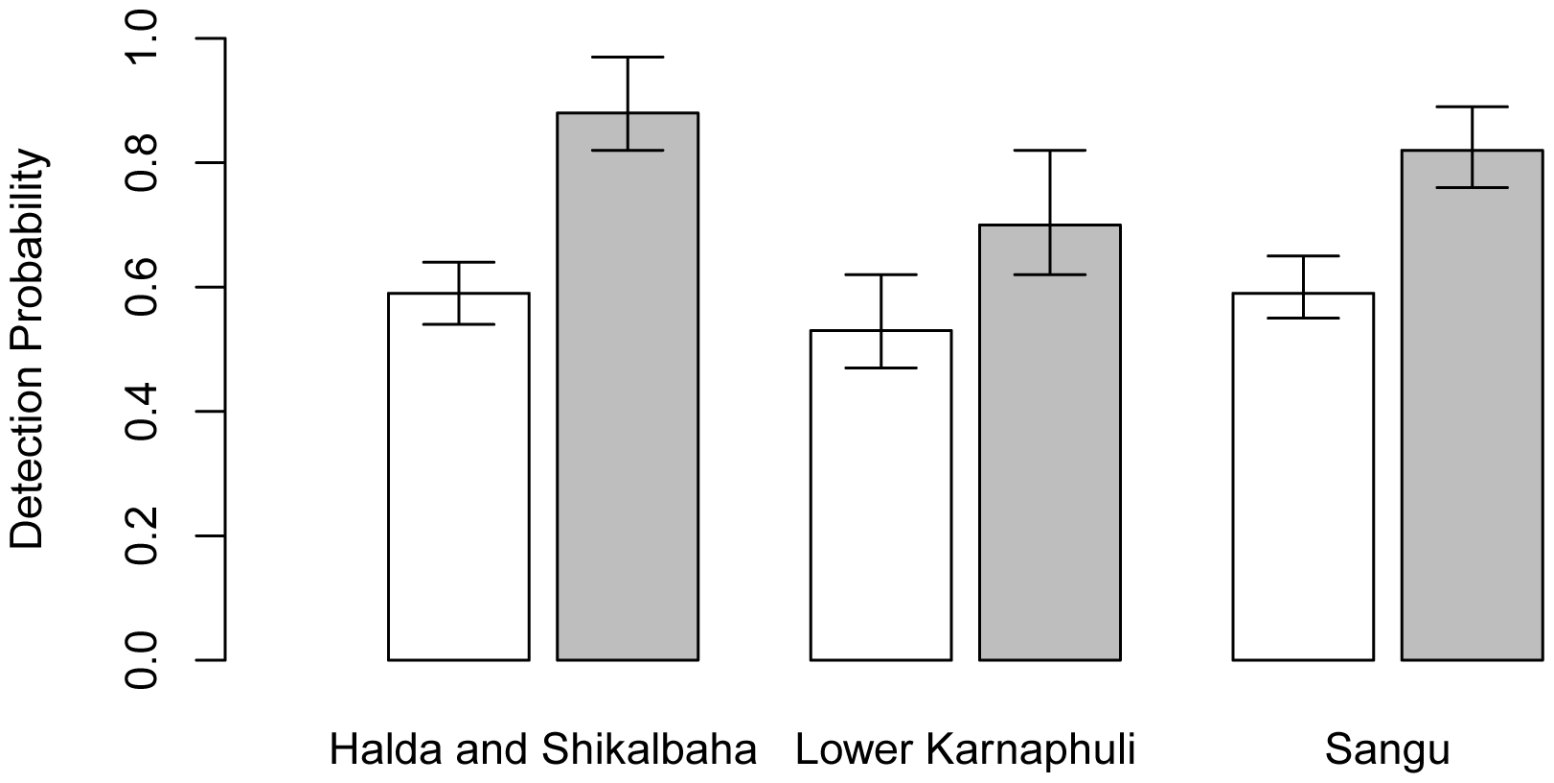
Detection probabilities and 95% confidence intervals for visual (white) and acoustic (light grey) methods.

Acoustic detectability was consistently higher than visual detectability (Figure 4) with notable differences in estimates between the Halda and Shikalbaha-Chandkhali Canal. Both acoustic and visual detectability were lowest in the Lower Karnaphuli River, but overall there was little difference in estimates between the rivers. In the Upper Karnaphuli River four individuals detected acoustically were not detected visually. Overall visual and acoustic detection probabilities were 0.57 (95% CI = 0.54–0.61) and 0.80 (95% CI = 0.75–0.85) respectively.

### Surveys Required to Detect Trends

The single observer-team visual surveys resulted in a lower survey strip population estimate with greater coefficient of variation (116: CV = 7%) than the combined visual-acoustic method (203: CV = 3%). If the population of 203 animals were to experience a decline of 24% between survey intervals, five survey repeats would be needed to detect a decline using the combined visual-acoustic method compared to nine survey repeats using the single observer method.

### Factors Affecting Detection by Observers

**Figure 5:**
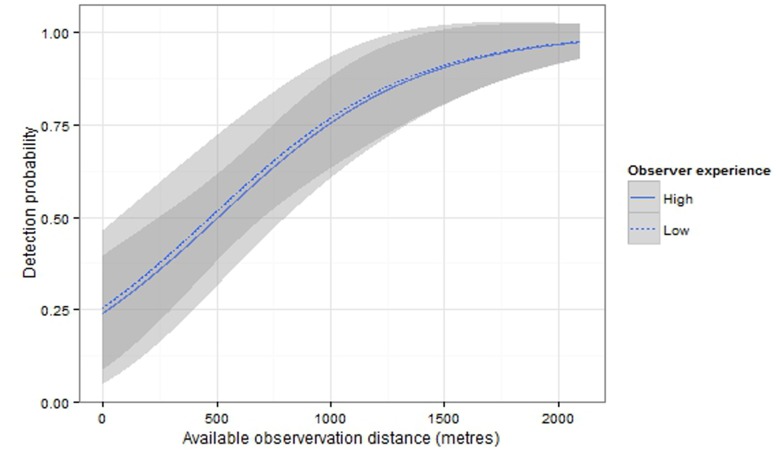
Predicted visual detectability and 95% confidence band, using model-averaged coefficients from candidate models.

There was no evidence of collinearity between any of the factors, and there was no strong support for a particular model as the top two models had Δ*_i_* ≤2 (Table 2). Coefficients (*β*) are averages of βi across the top two models, weighted by each model’s Akaike weight *ω_i_*. Model-averaged coefficients indicated that visual detectability was not significantly affected by observer experience (0.31, 95% CI = −0.48–1.1) but was affected by the available observation distance (0.0023, 95% CI = 0.0011–0.0035) (Figure 5). While there is considerable uncertainty in predicted values of visual detectability at available observation distances ≤500 m, visual detection probabilities were less than 0.5.

**Table 2:** Summary of models used to explore factors affecting visual detectability.

Available Observation Distance	Observer Experience	Interaction	K	AIC	Δ_i_	*w_i_*
Y	–	–	2	148.1	0	0.586
Y	Y	–	3	149.5	1.4	0.289
Y	Y	Y	4	151.2	3.1	0.122
–	Y	–	2	159.5	11.4	0.002

### Cost Analysis of Methods

Capital cost was highest for the combined visual-acoustic survey ($8,460) due to the cost of the hydrophone array ($8,000) (see Table S1 in the supplementary information). However, because of higher daily running costs, the tandem-vessel visual survey and double observer-team visual surveys exceeded the combined visual-acoustic survey in overall cost after 40 and 56 survey days respectively (see Figure S3 in the supplementary information). The single observer-team visual survey always remained the cheaper survey option as daily running costs were equivalent to the combined visual-acoustic survey.

## Discussion

The importance of accounting for imperfect detectability during wildlife surveys is widely recognised [62] but methods that fail to account for it remain in use for a range of taxa [4], [63]. Attempts have been made to account for imperfect detection during visual surveys of freshwater cetaceans by using double observer-team visual surveys e.g. [24] or tandem-vessel visual surveys e.g. [25], but given that these methods are often impractical and do not account for availability bias, new approaches are needed. In this study we use a novel method for estimating abundance of Ganges River dolphins that accounts for imperfect detectability and improves the precision of abundance estimates. Our results show that acoustic detectability is consistently greater than visual detectability because animals can be detected when submerged [7], [25], thereby reducing availability bias which can be a significant problem for visual surveys of diving animals.

### Availability Bias

Evaluations of availability bias for diving animals are typically undertaken by calculating the number of potential surfacings within the visual range of observers for a given boat speed [23], [24]. Dive time in marine mammals can be affected by physiological factors, such as oxygen storage and consumption [64], as well as external factors such as presence of vessel traffic [65], [66]. A recent study of Ganges River dolphins [47] found that time of day did not significantly affect dive time, although this study was based on limited survey effort. Mean estimates of dive time for Ganges River dolphins are typically in the range of 70–115 seconds [24], [46], [47], although two studies have recorded dive times as high as 465 seconds and 504 seconds [47], [66]; such long dive times would greatly decrease visual availability. Unless studies adequately capture the factors that can affect dive time, then estimates from observations of a few groups may not adequately represent the distribution of likely dive times, resulting in biased estimates of abundance as assumptions about availability remaining consistent across surveys may not be met.

### Perception Bias

Visual barriers (e.g. meanders) may reduce the detectability of freshwater cetaceans, and such spatial variability in detectability may impact conclusions on habitat use [67]. Evidently in wide river systems (such as the main channels of the Sundarbans, Ganges and Brahmaputra) the negative effect of meanders on detectability would be minor as meanders would not significantly affect available observation distance. However, in narrow river channels, observation distance decreases substantially around a meander, thereby reducing the time available to view the following river surface and detect a potential surfacing. Our model shows that where available observation distance is less than 500 m, mean visual detection probabilities were less than 0.50. With a visual range of 500 m, dolphins might surface only twice based on our mean estimates of dive time and a vessel speed of 10 km/hr. The use of a rear-facing observer or reducing boat speed around meanders may help overcome this bias, although reductions in boat speed may not be practical in high-velocity rivers. Without modifications to survey design, visual-only surveys may significantly underestimate population size in narrow, highly-meandering water ways, and therefore underestimate the importance of meanders as habitat for dolphins.

Studies have found that sighting rates of marine cetaceans differ significantly between experienced and inexperienced observers [68]. Increased observer experience is possibly associated with greater consistency in scanning behaviour when using binoculars [69], and particularly improved detection in adverse conditions. We did not find an effect of observer experience on detection probability. This may be due to the excellent sighting conditions throughout the survey, meaning that dolphin surfacings were almost always easily detectable. Furthermore, narrow river width meant that there was considerably less area for each observer to scan compared to observation in wide river systems or marine environments. A smaller area to scan may lessen the importance of effective search behaviour, as a greater proportion of the river surface is within the field of view.

### Trend Detection

Failure to explicitly account for biases [4] and low population density [70] can affect the ability to detect trends [71]. As a population declines, the minimum detectable rate of change tends to increase [70]. Notably, a study on the Vaquita, a highly threatened marine porpoise, showed that for a population size of 300, the minimum detectable rate of decline after ten annual distance sampling surveys was 18% [70]. Identification of methods that are able to detect declines quickly and with minimal effort is particularly important for Ganges River dolphins. While the global population may number in the thousands, the range of this species has been severely fragmented by the construction of dams [36], resulting in small isolated subpopulations [6]. Unless surveys can detect trends quickly, these subpopulations may fall below the minimum viable population size before a decline is detected. Under a ten year monitoring scheme, the combined visual-acoustic survey reduced the effort required to detect a rate of decline necessary for an IUCN listing of Critically Endangered under Criterion A. However, given the likely small size of many Ganges River dolphin subpopulations, we recommend that the goal of monitoring should be to detect declines in the shortest time frame possible to minimise the overall loss of individuals.

### Costs of Survey Methods

While acoustic surveys can reduce effort in terms of the number of repeat surveys required for trend detection, the capital costs of a hydrophone array and associated technical expertise remain a barrier to their wide-scale adoption in cetacean monitoring programmes. Limited resources encourage the use of low-cost, familiar methods for monitoring; however, unless detectability is accounted for this may prove a false economy if the goal is to detect trends [72]. Our results demonstrate that single observer-team visual surveys always remain a cheaper survey option but cannot account for detectability, and so despite this cost difference have limited value for monitoring. Despite the high capital cost of a combined visual-acoustic survey, lower running costs mean that relatively quickly it becomes the cheaper option out of the methods that do account for detectability, making it a cost-effective tool for monitoring. Through development of regional collaborations there could be the opportunity to share technical expertise and equipment, making acoustic surveys more practical for NGOs or governments wanting to carry out high-quality surveys.

### Limitations of Acoustic Surveys

While combined visual-acoustic surveys can overcome many of the availability and perception biases associated with visual surveys, factors affecting acoustic detectability are less well understood. Of the unmatched visual detections, most (20 of 23) were located either in Chittagong Port on the Lower Karnaphuli where there are considerable underwater barriers to acoustic detection created by ship hulls; or in high dolphin-density areas (visual group size >3) of the Sangu, where it is possible that observers overestimated group size. However, previous work suggests that accurate acoustic detection is negatively affected by higher dolphin densities [25] as it becomes difficult to visually distinguish individual click trains under such conditions. We acoustically detected a maximum of five individuals within any given distance window, but without knowing the true number of dolphins it is difficult to determine whether this was a limitation of data loggers or overestimation by visual observers.

Acoustic detectability declines over distance at a rate determined by the detection threshold of data loggers, the level of unwanted noise, and the source level of the phonating dolphin [25]. We were unable to determine the maximum acoustic detection range in our study area as animals were unevenly distributed across the river width. In the Yangtze River, an acoustic detection range of 300 m has been achieved for finless porpoises using the same hydrophone array as described here [25]. We expect it is possible to achieve a minimum detection range of 300 m in Ganges River dolphin habitat where noise levels are similar or less than the Yangtze, and source levels between species are comparable [27], [73].

While the sound beam of Ganges River dolphins is broad relative to other odontocetes, it is still relatively narrow and highly directional to facilitate prey discrimination in complex environments and under conditions of poor visibility [27]. Narrow beam width means that dolphins are only available for acoustic detection when oriented towards data loggers. While no acoustic studies have been carried out on the scanning behaviour of Ganges River dolphins, observations suggest that animals use changes in body orientation (e.g. side-swimming) and up-and-down head movements to increase their scan area [45]. These behaviours mean that dolphins are constantly changing orientation and are therefore likely to be detected acoustically despite the narrow beam.

Despite there being a range of factors that negatively affect acoustic detection, consistently higher estimates of acoustic detectability indicate that these factors exert less of an effect than the factors affecting visual detection. Furthermore, the advantage of combined visual-acoustic surveys become more apparent as populations decline as many of the factors affecting acoustic detection are unaffected by declining population size.

### Recommendations for Future Surveys

The recent uplisting of the Yangtze Finless porpoise from Endangered to Critically Endangered by IUCN [74], and the threatened status of most of the world’s other freshwater cetaceans, makes the identification of robust methods for estimating abundance for this group a priority. Single observer-team visual surveys are a relatively cheap, easy-to-implement method that has been widely used. If all factors affecting detectability could be kept constant, count data from these surveys could be treated as a relative index of abundance. However, many factors, some of which cannot be easily controlled, can affect detectability. For example, population declines can themselves affect detectability, so that any interpretation of trends in count data from visual surveys can be misleading [72].

There is growing evidence for the efficacy of combined visual-acoustic surveys as a monitoring tool for freshwater cetaceans. However, in order to optimise this method, future studies need to: focus on improving the matching of acoustic and visual detections; investigate whether the accuracy of acoustic counts is density-dependent; and investigate the variability in detection range for multiple species and how this is affected by variable levels of noise typically encountered in freshwater habitats.

### Conclusion

Freshwater cetaceans are one of the most threatened groups of mammals. Identification of robust methods for estimating population size and trend detection is therefore an important priority to accurately identify populations for conservation attention, and assess the effectiveness of management interventions. A range of methods are already used to try to achieve these aims, but they either do not account for imperfect detectability (single observer-team visual surveys), or are unsuitable in shallow river systems (double observer-team visual surveys which require large boats for two independent teams), or are very expensive and may not work well in some conditions (tandem-vessel visual survey where the two boats may have different fields of view). Combined visual-acoustic surveys can overcome many of the biases that negatively affect visual detection, thereby producing more precise and less biased estimates of abundance, and improved power to detect trends. We argue that barriers to acoustic surveys, such as technical expertise and cost, can be overcome through regional collaborations and sharing of equipment, making such surveys practical and cost-effective for NGOs or governments.

## Supporting Information

Figure S1 **Map of the southern rivers of Bangladesh in Chittagong District (Upper and Lower Karnaphuli River, Halda River, Sikalbaha-Chandkahli Canal, Sangu River).** The grey buffers indicate the river sections covered by the combined visual-acoustic survey and the vertical line shading represents the area of Chittagong Port along the Lower Karnaphuli River.
(TIF)

Figure S2 **Cumulative frequency distribution of acoustic (grey bars) and visual (white bars) detections over distance from the transect line.** Note that these data were only available for the Karnaphuli, Halda and Sangu rivers due to failure of one of the data loggers on day three.
(TIF)

Figure S3 **Overall cost of a single observer-team (thick black line), double observer-team (grey dotted line), tandem-vessel (thin black line) and combined visual-acoustic survey (thick dashed line) over number of survey days.**
(TIF)

Table S1 **A comparison of costs for four survey methods.**
(DOCX)
