# Supplementary figures and images for: To See or Not to See: Investigating Detectability of Ganges River Dolphins Using a Combined Visual-Acoustic Survey
Source: PLoS One. 2014 May 7;9(5):e96811. doi: 10.1371/journal.pone.0096811 (PMC4013050; doi:10.1371/journal.pone.0096811)

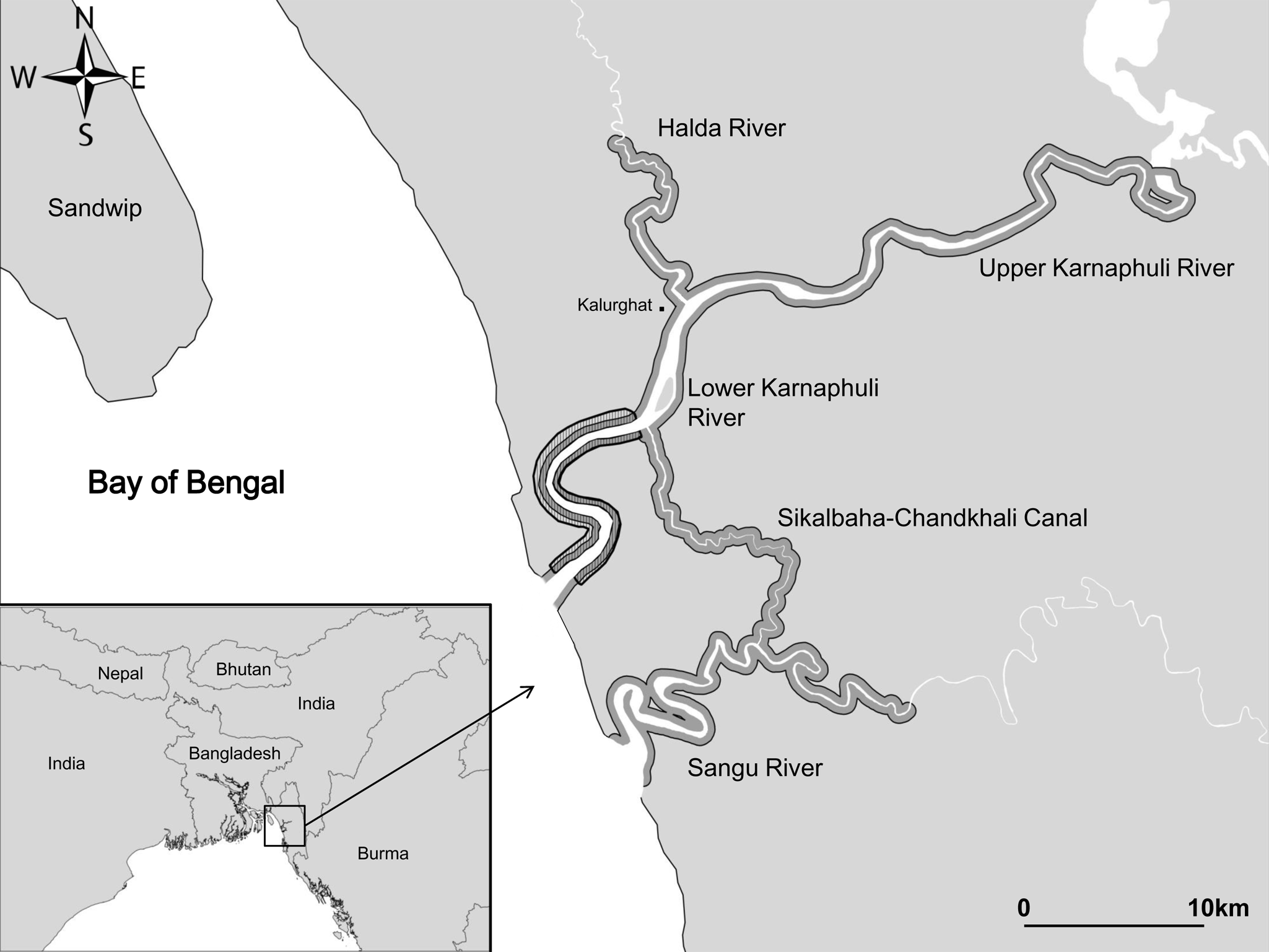

Supplement: Figure S1 — Map of the southern rivers of Bangladesh in Chittagong District (Upper and Lower Karnaphuli River, Halda River, Sikalbaha-Chandkahli Canal, Sangu River). The grey buffers indicate the river sections covered by the combined visual-acoustic survey and the vertical line shading represents the area of Chittagong Port along the Lower Karnaphuli River. (TIF) [file pone.0096811.s001.tif]

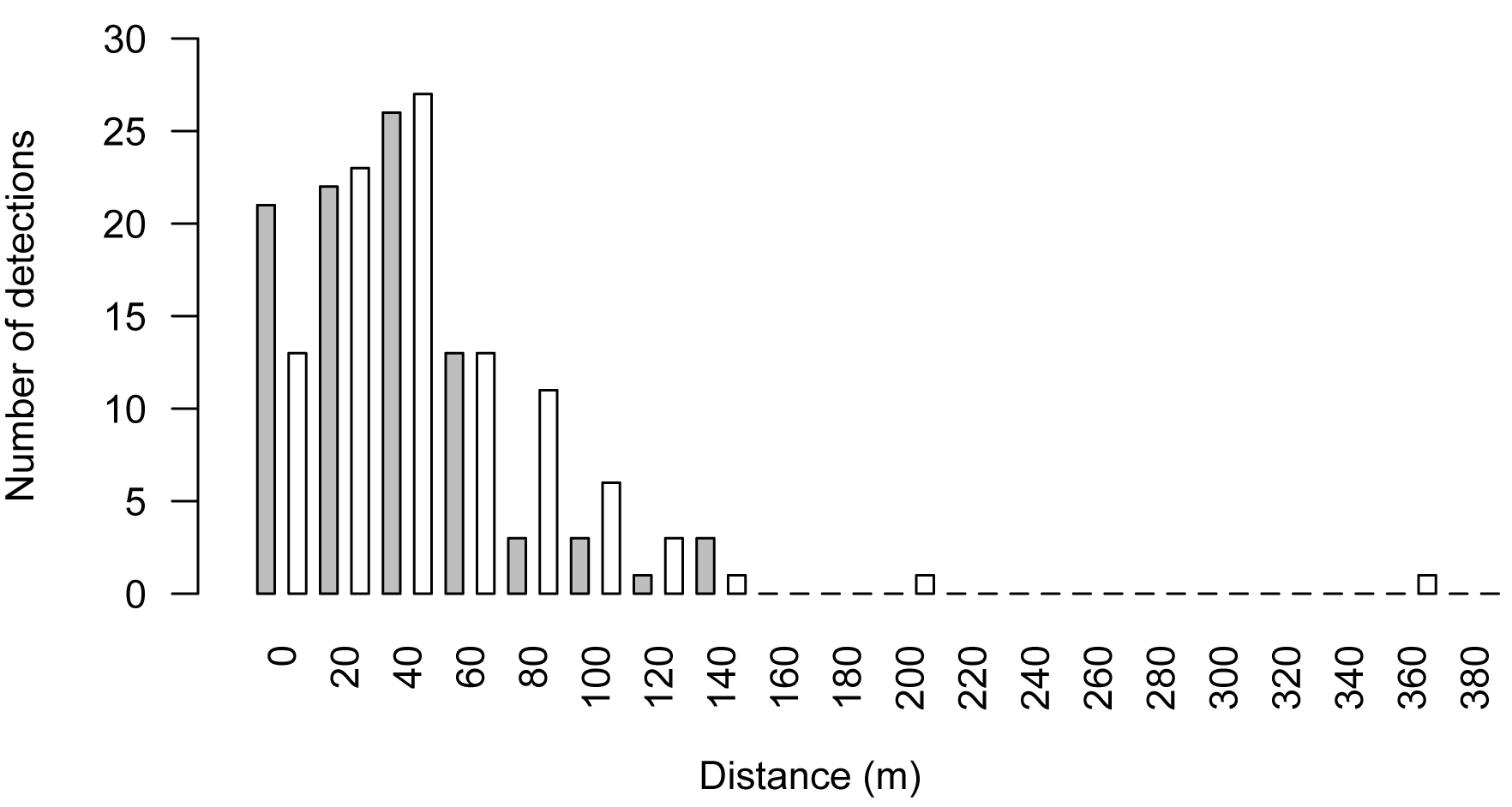

Supplement: Figure S2 — Cumulative frequency distribution of acoustic (grey bars) and visual (white bars) detections over distance from the transect line. Note that these data were only available for the Karnaphuli, Halda and Sangu rivers due to failure of one of the data loggers on day three. (TIF) [file pone.0096811.s002.tif]

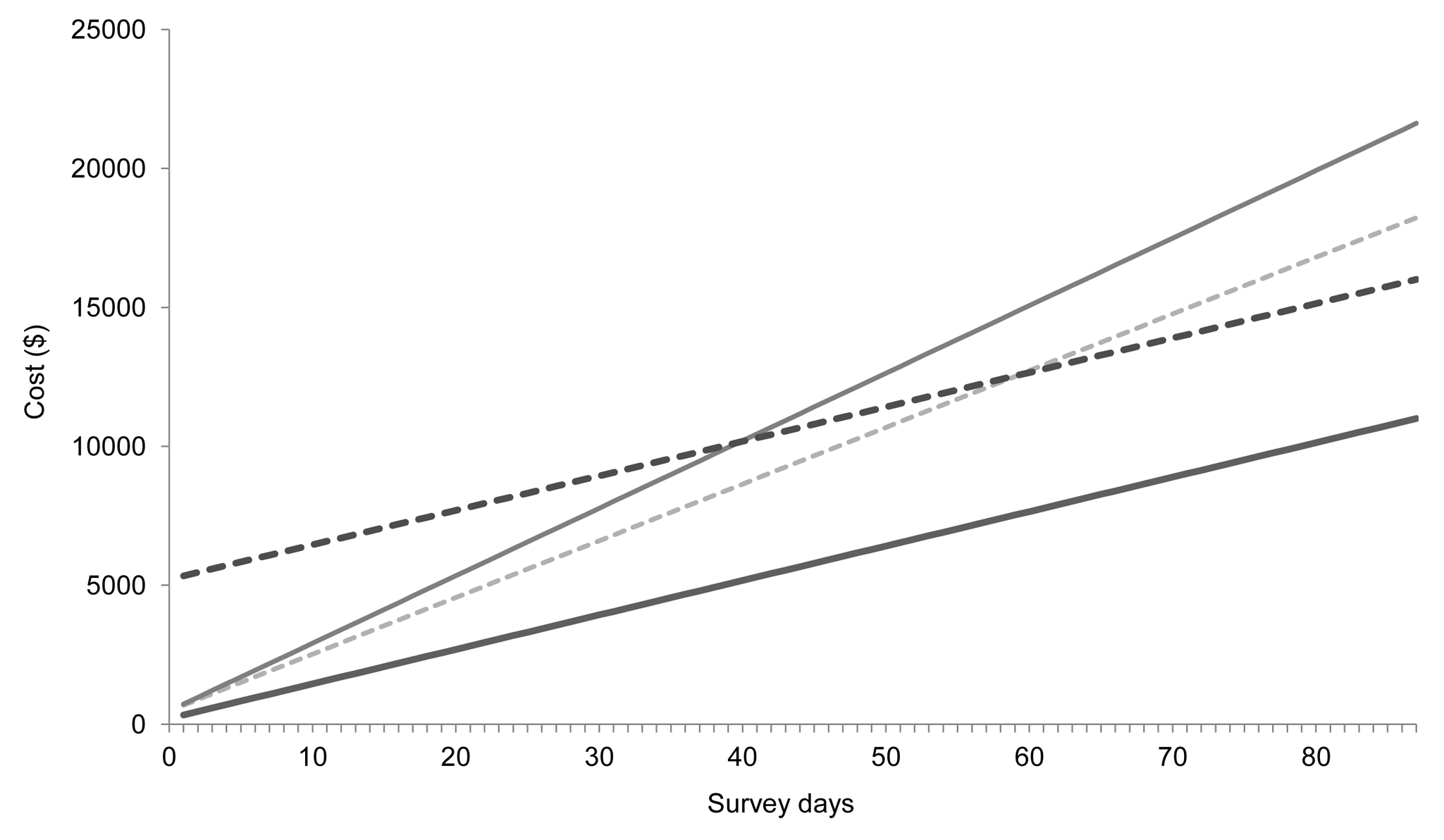

Supplement: Figure S3 — Overall cost of a single observer-team (thick black line), double observer-team (grey dotted line), tandem-vessel (thin black line) and combined visual-acoustic survey (thick dashed line) over number of survey days. (TIF) [file pone.0096811.s003.tif]
