# Supplementary material for: To See or Not to See: Investigating Detectability of Ganges River Dolphins Using a Combined Visual-Acoustic Survey
Source: PLoS One. 2014 May 7;9(5):e96811. doi: 10.1371/journal.pone.0096811 (PMC4013050; doi:10.1371/journal.pone.0096811)
Supplement: Table S1 — A comparison of costs for four survey methods. (DOCX) [file pone.0096811.s004.docx]

Table S1: A comparison of costs for four survey methods.

| Item | Single observer-team visual survey | | | Double observer-team visual survey | | | Tandem-vessel visual survey | | | Combined visual-acoustic survey | | |
| --- | --- | --- | --- | --- | --- | --- | --- | --- | --- | --- | --- | --- |
|  | Quantity | Capital | Daily Cost | Quantity | Capital | Daily Cost | Quantity | Capital | Daily Cost | Quantity | Capital | Daily Cost |
| Boat | 1 | - | $122 | 1 | - | $200 | 2 | - | $244 | 1 | - | $122 |
| Staff | 5 | - | $40 | 10 | - | $80 | 10 | - | $80 | 5 | - | $40 |
| Food and Water | 5 | - | $15 | 10 | - | $30 | 10 | - | $30 | 5 | - | $15 |
| Stationary | 1 | - | $8 | 1 | - | $8 | 1 | - | $8 | 1 | - | $8 |
| GPS | 1 | $110 | - | 2 | $200 | - | 2 | $220 | - | 2 | $220 | - |
| Binoculars | 3 | $240 | - | 6 | $480 | - | 6 | $480 | - | 3 | $240 | - |
| Hydrophone Array | - | - | - | - | - | - | - | - | - | 1 | $8000 | - |
| Total | **-** | **$350** | **$185** |  | **$700** | **$318** | **-** | **$700** | **$362** |  | **$8460** | **$185** |
